# GC/MS-based urine metabolomics analysis of renal allograft recipients with acute rejection

**DOI:** 10.1186/s12967-018-1584-6

**Published:** 2018-07-20

**Authors:** Long Zheng, Jina Wang, Wenjun Gao, Chao Hu, Shuo Wang, Ruiming Rong, Yinlong Guo, Tongyu Zhu, Dong Zhu

**Affiliations:** 1 Department of Urology, Zhongshan Hospital, Fudan University, Shanghai, 200032 China; 2 Shanghai Key Laboratory of Organ Transplantation, 180 Fenglin Road, Shanghai, 200032 China; 3 Department of Blood Transfusion, Zhongshan Hospital, Fudan University, Shanghai, 200032 China; 4 National Center for Organic Mass Spectrometry in Shanghai, Shanghai Institute of Organic Chemistry, Chinese Academy of Sciences, 345 Lingling Road, Shanghai, 200032 China; 5 Department of Urology, Qilu Hospital of Shandong University, Jinan, 250000 Shandong China

**Keywords:** GC/MS, Urine metabolites, Acute rejection, Renal transplantation

## Abstract

**Background:**

Acute renal allograft rejection is a common complication after renal transplantation that often leads to chronic rejection and ultimate graft loss. While renal allograft biopsy remains the gold standard for diagnosis of acute rejection, the possibility of biopsy-associated complications cannot be overlooked. The development of noninvasive methods for accurate detection of acute renal allograft rejection is thus of significant clinical importance.

**Methods:**

Gas chromatography–mass spectrometry (GC/MS) was employed for analysis of urine metabolites in 15 renal allograft recipients with acute rejection and 15 stable renal transplant recipients. Partial least squares (PLS) regression and leave-one-out analyses were performed to ascertain whether the metabolites identified could be exploited to distinguish acute rejection from stable groups as well as their sensitivity and specificity.

**Results:**

Overall, 14 metabolites were significantly altered in the acute rejection group (11 and 3 metabolites displayed higher and lower levels, respectively) relative to the stable transplant group. Data from PLS and leave-one-out analyses revealed that the differential metabolites identified not only distinguished acute rejection from stable transplant recipients but also showed high sensitivity and specificity for diagnosis of renal allograft recipients with acute rejection.

**Conclusion:**

Urine metabolites identified with GC/MS can effectively distinguish acute rejection from stable transplant recipients, supporting the potential utility of metabolome analysis in non-invasive diagnosis of acute rejection.

## Background

Renal transplantation is commonly recognized as an effective therapy for patients with end-stage renal disease (ESRD) [1]. Despite substantial improvements in immunological matching, surgical techniques and immunosuppressive drugs, recipients continue to experience post-operational complications that damage survival of both renal allografts and recipients. Acute rejection, a common complication of renal allograft, is considered an important risk factor for chronic rejection and ultimate graft loss, especially when repeated episodes occur [2–4]. However, clinical signs are insufficient to distinguish acute rejection from other causes of renal allograft dysfunction, such as drug toxicity, bacterial or viral infection. While renal allograft biopsy remains the gold standard for diagnosis of acute rejection, the risk of biopsy-associated complications, including haematuria, anuria, perirenal haematoma, bleeding, shock, arteriovenous fistula and graft loss, remains unavoidable [5]. Sampling errors in biopsies may additionally result in subsequent disparities between clinical and microscopic findings. Noninvasive methods to accurately detect acute renal allograft rejection are therefore an urgent clinical requirement.

Metabolomics, characterized by high-throughput and quantitative measurement of all small-molecule metabolites in the metabolome, targets the similarities and differences between biological samples [6]. This technique has been used in a number of areas, including identification of potential biomarkers of disease, pharmaceutical research, nutrition and botanical science [7]. Multiple analytical approaches have been applied for metabolomic analyses, including gas chromatography–mass spectrometry (GC/MS), liquid chromatography–mass spectrometry (LC–MS) and proton nuclear magnetic resonance (NMR) [8–10]. In particular, GC/MS has been extensively applied in the identification and quantification of metabolites owing to several advantageous features, such as higher resolution and sensitivity and availability of databases [11]. In combination with bioinformatic and biostatistical analyses, this technology may contribute to the identification and quantification of small-molecule metabolites to characterize whole-organism response to a given disease.

In the present study, GC/MS was performed to compare small-molecule metabolites in urine of recipients with acute renal allograft rejection and stable kidney function, with the aim of: (1) identifying and characterizing specific urine metabolite profiles in renal allograft patients with acute rejection, (2) investigating the sensitivity and specificity of small-molecule metabolites in diagnosis of patients with acute renal allograft rejection, and (3) providing novel biomarkers to facilitate the identification of potential patients with acute renal allograft rejection and improve current diagnostic methods and standards.

## Methods

### Patients and sample collection

In total, 30 urine samples were collected from 15 transplant recipients with acute rejection and 15 stable renal allograft recipients. All cases of acute rejection were confirmed via biopsy of specimens evaluated by an independent, blinded pathologist. Biopsy-validated acute rejection was based on the Banff 97 classification criteria. Urine was collected prior to renal allograft biopsy using 50 ml sterile polypropylene centrifuge tubes. After centrifugation at 3000 rpm for 10 min at 20 °C, supernatant fractions were collected and stored at − 80 °C until use.

### Preparation of urine samples

Urine samples were thawed at 4 °C and centrifuged at 10,000 rpm for 5 min. The supernatant (200 µl) was transferred to a 2 ml Eppendorf tube along with 20 µl urease (30 U) before incubation at 37 °C for 20 min. An aliquot of urine (200 µl) was extracted using 1600 µl methanol and deionized water, followed by centrifugation at 13,000 rpm for 10 min at 4 °C. The supernatant (1700 µl) was transferred for evaporation under a stream of N_2_ gas until dryness. After adding 30 µl methoxypyridine (15 µg/µl), the resultant mixture was mixed via vortexing for 2 min and methoxymation conducted at room temperature for 16 h. Subsequently, 30 µl methyl-*N*-trimethylsilyltrifluoroacetamide (MSTFA) with 1% chlorotrimethylsilane (TMCS) was added and mixed via vortexing for 2 min. After silylation for 30 min, 40 µl heptane was added, the mixture centrifuged at 10,000 rpm for 5 min, and 80 µl supernatant transferred for GC/MS analysis.

### GC/MS analysis

An aliquot (1 µl) of each derivatized sample was injected splitlessly using an Agilent 7683 autosampler (Agilent, Atlanta, GA) into an Agilent 7890A gas chromatograph. The injector temperature was 280 °C and gas flow rate through the column was 1 ml/min. The GC temperature program was set as follows: 70 °C for 2 min, a temperature ramp of 20 °C/min up to 140 °C, a second temperature ramp of 5 °C/min up to 200 °C (held for 1 min), and a third temperature ramp of 30 °C /min up to 300 °C (held for 20 min). The temperature of the transfer line was set at 250 °C. Full-scan EI spectra were acquired under the following conditions: scan time 1 s, mass range 50–650 m/z, ion trap temperature 200 °C, solvent delay 240 s, and emission current 2 mA (at 70 eV electron energy).

### Data processing

The acquired chromatograms were imported into AMDIS version 2.0 (NIST, USA). Subsequently, noise analysis, component perception and spectral deconvolution were performed. The components recognized were subjected to a NIST library search. Components with match factor > 80% were selected manually to construct the urine metabolome for each patient and those with match factor < 80% removed.

### Statistical analysis

#### Clinical data analysis

Data on gender, age, HLA mismatch and immunosuppressant regimens are expressed as mean ± standard deviation (SD). SPSS software version 19 (IBM SPSS, Armonk, NY, USA) was employed to analyze data for performance of the t test. Data were considered significantly different at P < 0.05.

### Metabolic analysis

The metabolite signal value was compared with the external standard signal value for removal of systematic errors. The t test was used to analyze statistical differences between the groups of metabolites. The VB 6.0 self-compiled program was used to reduce the dimensions of data and extract features. Subsequently, partial least squares (PLS) analysis was performed to detect whether the identified metabolites could distinguish between the acute rejection and stable transplant groups. Leave-one-out statistical analysis was applied to establish predictive models to estimate the sensitivity and specificity of the differential metabolites identified. Briefly, 30 samples were randomly divided into five groups (six per group), among which four groups were used for model establishment to predict the remaining sample groups. Subsequently, the remaining group was returned to the pool while another group of samples was drawn. A new model was established using the new four-group samples to predict the newly drawn group. This process was repeated five times. Using 0.44 as the selected dividing value, the total number of misclassified samples was calculated and the error rate finally estimated.

## Results

### Clinical data analysis

Comparative analysis of several factors, including age, gender, HLA typing, immunosuppressive regime and kidney source, revealed no significant differences, as shown in Table 1. Detailed information on the 15 renal allograft recipients with acute rejection and 15 stable renal allograft recipients is provided in Tables 2 and 3. The 15 stable renal allograft recipients recruited included 10 males and 5 females with an average age of 32.9 ± 13.0 years and HLA mismatch of 1.73 ± 1.03. Overall, 13 patients underwent immunosuppressive therapy involving cyclosporin (CsA)+mycophenolate mofetil (MMF)+Prednisone (Pred) and the two remaining patients were administered tacrolimus (FK)+MMF+Pred. Twelve cases were obtained from living donors and 3 from donors that underwent cardiac death. The 15 renal allograft recipients with acute rejection included 12 males and 3 females with an average age of 35.9 ± 10.7 years and HLA mismatch of 1.87 ± 0.99. Within this group, 11 patients were subjected to CsA+MMF+Pred and 4 prescribed FK+MMF+Pred. Eleven donations were from living patients and 4 from donors that underwent cardiac death.

**Table 1:** Clinical data of renal allograft recipients in the acute rejection and stable groups

	Acute rejection group (n = 15)	Stable group (n = 15)
Gender (M/F)	(12/3)	(10/5)
Age	35.9 ± 10.7	32.9 ± 13.0
HLA mismatch	1.87 ± 0.99	1.73 ± 1.03
Immunosuppressant regiments		
CsA+MMF+Pred	11	13
FK+MMF+Pred	4	2
Living donor	11	12
Donor of cardiac death	4	3

**Table 2:** Detailed clinical data of acute renal allograft rejection recipients

Recipients	Gender	Age	HLA mismatch	PRA	Immunosuppressant regiments	Pathological grade	Postoperative time	Serum creatinine (μmol/L)
AR1	M	23	2/6	–	CsA+MMF+Pred	III	14 days	161
AR2	M	23	2/6	–	CsA+MMF+Pred	II	2 min	210
AR3	M	44	1/6	–	FK+MMF+Pred	I	10 days	183
AR4	M	33	0/6	–	CsA+MMF+Pred	I	5 min	168
AR5	F	31	1/6	–	CsA+MMF+Pred	I	9 min	126
AR6	M	29	1/6	–	CsA+MMF+Pred	III	3 min	128
AR7	M	29	3/6	–	CsA+MMF+Pred	I	3 years	125
AR8	M	45	3/6	–	FK+MMF+Pred	III	5 min	151
AR9	F	55	3/6	–	CsA+MMF+Pred	I	6 min	99
AR10	M	45	3/6	–	CsA+MMF+Pred	I	3 years	135
AR11	F	20	1/6	–	FK+MMF+Pred	I	3 years	126
AR12	M	43	3/6	–	CsA+MMF+Pred	I	2 years	122
AR13	M	48	1/6	–	FK+MMF+Pred	I	50 days	166
AR14	M	42	2/6	–	CsA+MMF+Pred	I	7 min	159
AR15	M	28	2/6	–	CsA+MMF+Pred	I	5 min	208

**Table 3:** Detailed clinical data of stable renal allograft recipients

Recipients	Gender	Age	HLA mismatch	PRA	Immunosuppressant regiments	Pathological grade	Postoperative time (days)	Serum creatinine (μmol/L)
S1	M	25	2/6	–	CsA+MMF+Pred	Normal	15	94
S2	F	54	1/6	–	FK+MMF+Pred	Normal	15	59
S3	M	32	2/6	–	CsA+MMF+Pred	Normal	15	94
S4	M	32	1/6	–	CsA+MMF+Pred	Normal	15	103
S5	F	55	3/6	–	CsA+MMF+Pred	Normal	15	99
S6	M	39	2/6	–	CsA+MMF+Pred	Normal	15	65
S7	F	27	2/6	–	CsA+MMF+Pred	Normal	15	88
S8	F	6	0/6	–	CsA+MMF+Pred	Normal	15	56
S9	M	25	1/6	–	CsA+MMF+Pred	Normal	15	67
S10	M	17	0/6	–	FK+MMF+Pred	Normal	15	111
S11	M	23	3/6	–	CsA+MMF+Pred	Normal	15	68
S12	M	19	1/6	–	CsA+MMF+Pred	Normal	15	106
S13	M	30	2/6	–	CsA+MMF+Pred	Normal	15	85
S14	M	31	3/6	–	CsA+MMF+Pred	Normal	15	98
S15	F	58	3/6	–	CsA+MMF+Pred	Normal	15	108

### Metabolic profiling

**Fig. 1:**
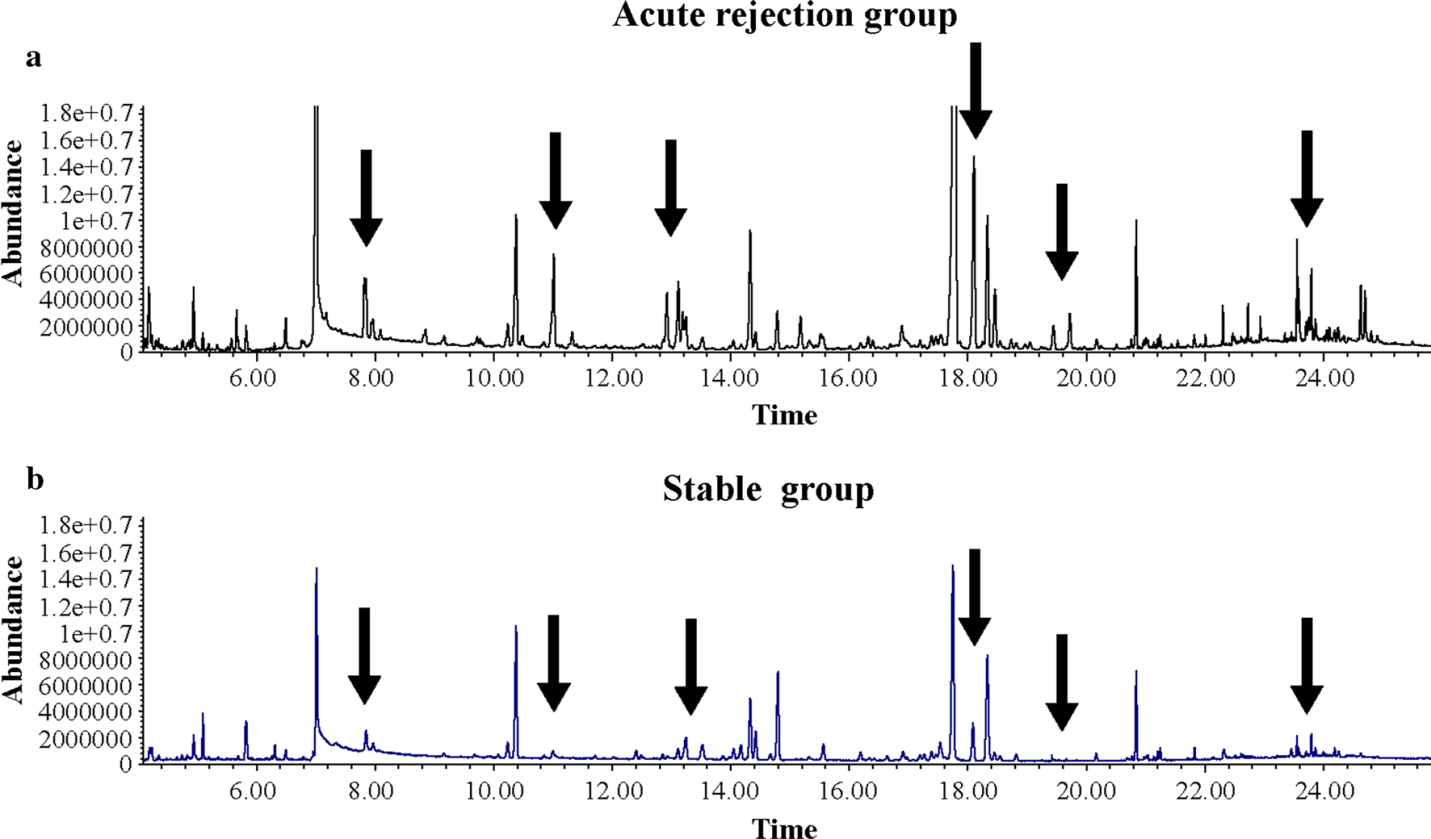
Representative serum total ion current mass spectra of samples from acute renal allograft rejection and stable recipients. **a** Urine samples from acute renal allograft rejection recipients. **b** Urine samples from stable recipients. The arrows indicate markedly different wave crests between **a**, **b**

In a typical GC/MS analysis, nearly 200 small-molecule metabolites with a mass-to-charge ratio of 50–650 Da were detected. As shown in Fig. 1, significant differences in the total ion chromatogram of the urine sample were evident between renal allograft recipients with acute rejection and stable renal allograft function. The metabolites were identified on the grounds of similarity between the determined mass spectrometry and standard mass spectrometry in the NIST database, and those showing repeatability in the intra- and inter-day assay selected for further bioinformatics analysis. Non-endogenous metabolites, such as drugs and reagents, were manually excluded.

Using the t-test to compare metabolite contents between the acute rejection and stable groups, we identified 14 metabolites that were significantly different, namely threitol, inositol, glucose, fructose, xylono-1, 5-lactone, xylitol, xylopyranoside, 2,3-dihydroxybutanoic acid, glucitol, ribonic acid, glycolic acid, 3-hydroxyisovaleric acid, octadecanoic acid and phosphate (Table 4). Among these, the contents of 11 metabolites were significantly higher in the acute rejection than the stable group, specifically, threitol (0.126 ± 0.0625 vs. 0.0694 ± 0.0311, P < 0.05), inositol (0.127 ± 0.126 vs. 0.0458 ± 0.0379, P < 0.05), glucose (0.0263 ± 0.0113 vs. 0.0182 ± 0.00713, P < 0.05), xylono-1, 5-lactone (0.0291 ± 0.0193 vs. 0.0169 ± 0.00770, P < 0.05), xylitol (0.399 ± 0.167 vs. 0.288 ± 0.0892, P < 0.05), xylopyranoside (0.0769 ± 0.00478 vs. 0.0465 ± 0.0212, P < 0.05), 2,3-dihydroxybutanoic acid (0.0897 ± 0.0621 vs. 0.0496 ± 0.0307, P < 0.05), glucitol (0.201 ± 0.146 vs. 0.113 ± 0.0729, P < 0.05), ribonic acid (0.0548 ± 0.0324 vs. 0.0131 ± 0.0170, P < 0.001), octadecanoic acid (0.0171 ± 0.0116 vs. 0.0079 ± 0.0085, P < 0.001) and phosphate (2.21 ± 1.30 vs. 0.826 ± 0.0182, P < 0.001). The levels of three metabolites were markedly lower in the acute rejection than the stable transplant group, specifically fructose (0.0157 ± 0.00132 vs. 0.0325 ± 0.0251, P < 0.05), glycolic acid (0.0498 ± 0.0219 vs. 0.119 ± 0.0575, P < 0.001) and 3-hydroxyisovaleric acid (0.0151 ± 0.0109 vs. 0.0366 ± 0.0182, P < 0.001).

**Table 4:** List of metabolites and significant differences between two groups

Metabolite	Stable kidney function	Acute rejection	P value
Carbohydrate			
Threitol	0.0694 ± 0.0311	0.126 ± 0.0625	< 0.05
Inositol	0.0458 ± 0.0379	0.127 ± 0.126	< 0.05
Glucose	0.0182 ± 0.00713	0.0263 ± 0.0113	< 0.05
Fructose	0.0325 ± 0.0251	0.0157 ± 0.0132	< 0.05
Xylono-1,5-lactone	0.0169 ± 0.00770	0.0291 ± 0.0193	< 0.05
Xylitol	0.288 ± 0.0892	0.399 ± 0.167	< 0.05
Xylopyranoside	0.0465 ± 0.0212	0.0769 ± 0.00478	< 0.05
2,3-Dihydroxybutanoic acid	0.0496 ± 0.0307	0.0897 ± 0.0621	< 0.05
Glucitol	0.113 ± 0.0729	0.201 ± 0.146	< 0.05
Carboxylic acid			
Ribonic acid	0.0131 ± 0.0170	0.0548 ± 0.0324	< 0.001
Glycolic acid	0.119 ± 0.0575	0.0498 ± 0.0219	< 0.001
3-Hydroxyisovaleric acid	0.0366 ± 0.0182	0.0151 ± 0.0109	< 0.001
Octadecanoic acid	0.0079 ± 0.0085	0.0171 ± 0.0116	< 0.05
Others			
Phosphate	0.826 ± 0.0182	2.21 ± 1.30	< 0.001

Metabolite level was compared by T test for fold change and significant differences. P value was listed when P < 0.05. *FC* fold change (AR vs SG)

### Diagnostic sensitivity and specificity of the 14 metabolites

**Fig. 2:**
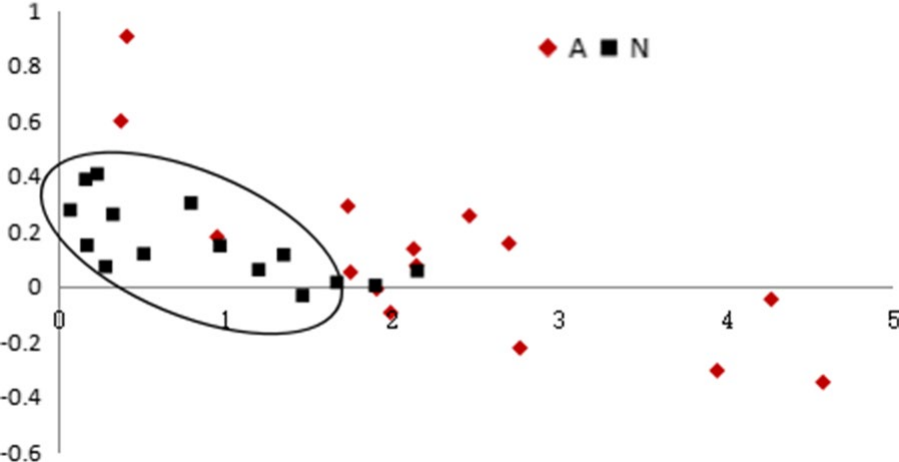
PLS analysis of 14 metabolites in urine samples of acute renal allograft rejection and stable recipients. Red, acute renal allograft rejection recipients; blue, stable recipients

PLS results showed that urine metabolites in the acute rejection and stable groups were separately clustered, supporting their potential ability to distinguish between the two patient groups (Fig. 2). Leave-one-out analysis was further performed to examine the sensitivity and specificity of the 14 metabolites. As shown in Table 5, with selection of 0.44 as the dividing value, 13 out of 15 renal allograft recipients in the acute rejection group and 10 out of 15 renal allograft recipients with stable kidney function were accurately diagnosed. Diagnostic sensitivity and specificity were estimated as 86.7 and 67.7%, respectively.

**Table 5:** Statistical efficiency based on leave-one-out analysis for 14 different urine metabolites

	Acute rejection group	Stable group
1	0.441414	0.364503
2	0.923023	0.462737^a^
3	0.612142	0.378701
4	0.837623	0.410927
5	1.022192	0.443031^a^
6	0.384961^a^	0.250744
7	0.860957	0.680434^a^
8	0.34546^a^	0.226024
9	0.514041	0.257583
10	0.493134	0.128227
11	0.525911	0.36553
12	0.470761	0.509088^a^
13	0.491634	0.162171
14	0.667121	0.563451^a^
15	1.076222	0.400769

^a^Samples predicted incorrectly with leave-one-out analysis model

### Diagnostic sensitivity and specificity of inositol, phosphate and octadecanoic acid

**Fig. 3:**
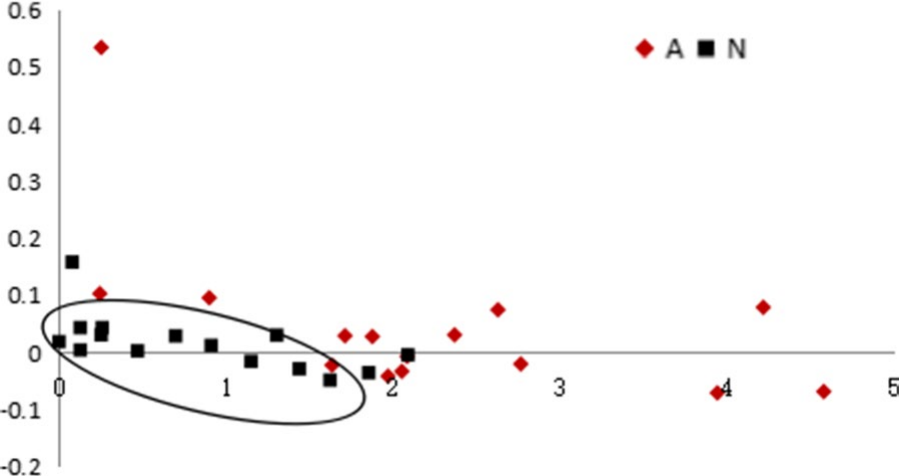
PLS analysis of urine inositol, phosphate and octadecanoic acid in acute renal allograft rejection and stable recipients. Red, acute renal allograft rejection recipients; blue, stable recipients

PLS and leave-one-out analysis were further employed analyze inositol, phosphate, and octadecanoic acid, which are involved in T cell activation. As shown in Fig. 3, plots representing the acute rejection group did not overlap with those of the stable transplant group, implying clear differentiation between the two groups based on these three metabolites. Leave-one-out data showed that 12 out of 15 renal allograft recipients in both acute rejection and stable kidney function groups were correctly diagnosed (Table 6), indicative of satisfactory diagnostic sensitivity and specificity.

**Table 6:** Statistical efficiency based on leave-one-out analysis for inositol, phosphate and octadecanoic acid

	Acute rejection group	Stable group
1	0.460188	2.45E−01
2	1.236908	0.219132
3	0.444361	3.47E−02
4	0.68486	0.416385
5	1.501095	0.427249
6	0.529902	1.89E−04
7	0.824111	0.655542^a^
8	0.244092^a^	0.127944
9	7.02E−02^a^	7.31E−02
10	0.614927	8.49E−02
11	0.560296	2.07E−02
12	7.64E−02^a^	0.509639^a^
13	0.75128	3.50E−02
14	0.594007	0.536628^a^
15	1.381454	0.377255

^a^Samples predicted incorrectly with leave-one-out analysis model

### Diagnostic sensitivity and specificity of ribonic acid, glycolic acid, 3-hydroxyisovaleric acid and octadecanoic acid

**Fig. 4:**
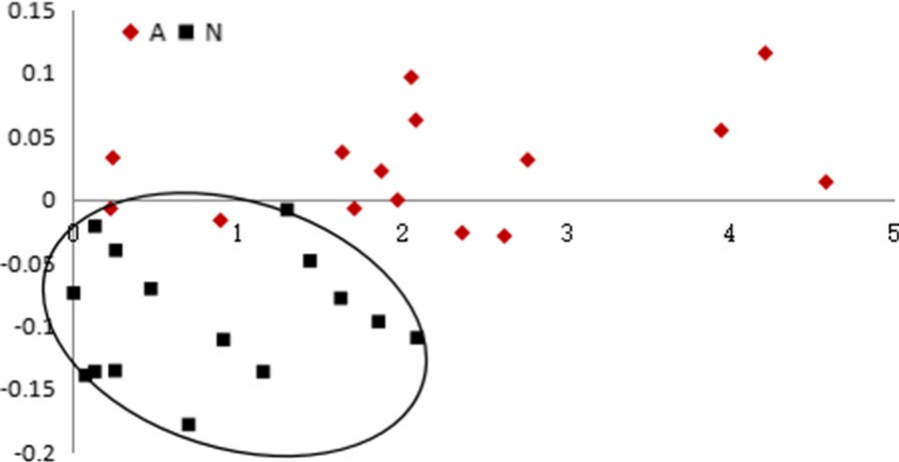
PLS analysis of ribonic acid, glycolic acid, 3-hydroxyisovaleric acid and octadecanoic acid in acute renal allograft rejection and stable recipients. Red, acute renal allograft rejection recipients; blue, stable recipients

The same analytical methods were applied for metabolites with P values < 0.001, specifically, ribonic acid, glycolic acid, 3-hydroxyisovaleric acid and octadecanoic acid. In PLS analysis, plots of the acute rejection group did not overlap with those of the stable group (Fig. 4), validating the utility of these 4 metabolites in distinguishing acute rejection from stable transplant patients. In leave-one-out analysis, 12 out of 15 renal allograft recipients in the acute rejection group and 13 out of 15 recipients possessing stable kidney function were correctly diagnosed. The diagnostic sensitivity and specificity were 80 and 86.7%, respectively (Table 7).

**Table 7:** Statistical efficiency based on leave-one-out analysis for ribonic acid, glycolic acid, 3-hydroxyisovaleric acid and octadecanoic acid

	Acute rejection group	Stable group
1	0.459	0.245336
2	1.235464	0.220303
3	0.444057	3.58E−02
4	0.684315	0.416607
5	1.497874	0.425581
6	0.529507	7.34E−04
7	0.822096	0.65566^a^
8	0.243125^a^	0.128246
9	6.56E−02^a^	7.38E−02
10	0.613041	0.08476
11	0.559469	0.020265
12	7.55E−02^a^	0.51026
13	0.7501	3.58E−02
14	0.594403	0.537187^a^
15	1.376774	0.378446

^a^Samples predicted incorrectly with leave-one-out analysis model

## Discussion

Owing to rapid developments in immunological matching, surgical techniques and immunosuppressive drugs, renal transplantation has been employed as the preferred treatment type for patients with ESRD over the past decade. However, acute renal allograft rejection remains a vital determining factor for short-term function and long-term outcome of both recipients and allografts. Due to multiple complications in renal biopsy, identification of effective rejection-related biomarkers independent of invasive biopsy in acute rejection patients is of significant clinical importance. Fifteen biopsy-proven acute rejection patients and 15 normal transplant recipients verified based on pathological analyses were enrolled in the present study. Comparison of multiple factors, including age, gender, HLA typing, immunosuppressive regime and kidney source, between the two groups revealed no significant differences. Analysis of urine metabolites via GC/MS disclosed marked alterations in 14 metabolites in the acute rejection group, compared with the stable group. Among these, nine were carbohydrates, four carboxylic acid and one mineral acid, indicating significant differences in metabolite profiling between the acute rejection and stable groups.

Acute renal allograft rejection is characterized by recruitment of activated leukocytes into the renal allograft, which is the basis for conventional Banff classification [12, 13]. Activated leukocytes exhibit high metabolic activity and metabolite measurements may therefore provide certain clues for diagnosis of acute rejection of renal allografts. Earlier, Kentrup et al. reported the utility of glucose chemical exchange saturation transfer (glucoCEST) magnetic resonance imaging in monitoring the d-glucose content in kidney. Using this novel method, the group showed that renal allografts undergoing acute rejection present significantly elevated glucose metabolism in transplanted rats, compared to healthy controls, ischemia reperfusion injury (IRI) and syngeneic renal transplantation rat models [14]. Measurement of the glucose analog, fluorodeoxyglucose F^18^ (^18^F-FDG), with positron emission tomography (PET) by Reuter et al. [15] revealed that renal ^18^F-FDG uptake is obviously increased in allogeneic transplanted rats with biopsy-proven acute rejection, compared to healthy controls, syngeneically transplanted rats and rats with acute cyclosporine nephrotoxicity, supporting a potential role of metabolism in acute renal allograft rejection. To establish whether metabolites can be utilized to distinguish between acute rejection and stable transplant groups, urine from 15 biopsy-proven acute rejection and 15 stable transplant recipients was collected and analyzed using GC/MS. The results showed that the levels of 11 metabolites were significantly higher (threitol, inositol, glucose, xylono-1, 5-lactone, xylitol, xylopyranoside, 2,3-dihydroxybutanoic acid, glucitol, ribonic acid, octadecanoic acid and phosphate) and three were markedly lower (fructose, glycolic acid and 3-hydroxyisovaleric acid) in the acute rejection relative to the stable transplant group.

In the present study, we aimed to identify the metabolites altered in response to acute rejection. GC/MS coupling, PLS and leave-one-out analyses were performed to determine sensitivity and specificity of the identified metabolites. The 14 metabolites that showed significant alterations between the groups could clearly distinguish acute rejection from stable transplant recipients. Overall diagnostic sensitivity and specificity of the 14 metabolites were 86.7 and 67.7% respectively, leading to diagnosis of 13 out of 15 renal allograft patients with acute rejection and 10 out of 15 with stable kidney function.

Acute renal allograft cellular rejection is a common T cell-mediated condition in kidney transplantation, which can perpetuate as chronic T cell-mediated rejection or transform into antibody-mediated rejection [16, 17]. Following interactions of the T cell antigen receptor (TCR) with major histocompatibility complex (MHC) molecules on the surface of antigen-presenting cells (APC), T cells are activated [18]. Upon binding, linker for activation of T cells (LAT) is phosphorylated by ZAP-70, in turn, leading to phosphorylation of tyrosine residues on the γ chain of membrane-bound phospholipase C (PLC-γ). Subsequently, phosphatidylinositol-4,5-bisphosphate (PIP2) is hydrolyzed into inositol triphosphate and diacylglcerol (DAG) by phosphorylated PLC-γ. DAG mediates activation of protein kinase C (PLC) while IP3 is involved in release of Ca^2+^ from intracellular stores, inducing activation of the Ca^2+^/calmodulin-dependent calcineurin. Calcineurin and PLC are responsible for activation of the transcription factors NF-AF and NF-kB, resulting in T cell activation and proliferation [19–21]. Inositol, phosphate and octadecanoic acid are the metabolites involved in this process. Our results showed that these three metabolites can distinguish between acute rejection and stable transplant groups. Moreover, PLS and leave-one-out analyses showed diagnostic sensitivity and specificity of up to 80%, facilitating correct diagnosis of 12 out of 15 renal allograft patients with acute rejection or normal pathology.

A total of four metabolites were identified based on P values < 0.001, supporting the utility of ribonic acid, glycolic acid, 3-hydroxyisovaleric acid and octadecanoic acid in distinguishing acute rejection from stable allograft recipients. Using PLS and leave-one-out analysis, diagnostic sensitivity and specificity were determined as 80 and 86.7% respectively, with accurate diagnosis of 12 out of 15 renal allograft patients with acute rejection and 13 out of 15 patients with stable kidney function.

Urine metabolites are potentially affected by various factors, including drugs and diseases. In an earlier study by Kadi et al. [22], rats administered masitinib (an oral drug for mast cell tumors) presented 20 more metabolites in the phase I metabolic pathway and 4 other metabolites in the phase II metabolic pathway, compared to control rats [22]. The group of Dawiskiba found that patients with active inflammatory bowel disease contain significantly lower levels of urine metabolites (citrate, hippurate, trigonelline, taurine, succinate and 2-hydroxyisobutyrate) than healthy controls [23], supporting the significance of urine metabolites in both pharmaceutical research and the clinical setting. In the present study, a total of 14 urine metabolites were identified that could effectively discriminate between acute rejection and stable transplant recipients. However, the number of urine samples examined was relatively small. We failed to precisely quantify the urine content of each metabolite and could not distinguish metabolites from functional metabolic pathways. Encouragingly, our data are concordant with the hypothesis that renal pathophysiologic changes are reflected by the urine metabolite content. Thus, monitoring of renal allograft function via GC/MS-based metabolomic analysis may show significant promise in helping to identify patients at high risk of acute allograft rejection.

## Conclusions

GC/MS-based identification of altered urine metabolites could be used to effectively distinguish between acute renal allograft rejection and stable transplant recipients. Moreover, PLS and leaving-one-out analysis revealed high sensitivity and specificity of the metabolites identified in diagnosis of renal allograft recipients with acute rejection, supporting the potential utility of metabolome analysis in non-invasive diagnosis of renal allograft rejection.
